# Stressors and coping strategies among Egyptian medical students under the integrated curriculum: a multicenter cross-sectional study

**DOI:** 10.1186/s12909-025-08487-8

**Published:** 2026-01-16

**Authors:** Mahmoud Abd El-Nasser, Aya Elsayed Abdelwahed, Nour Edin Darwish, Mohamed Rabiea Abdelnaby Fathallah Hamad, Aya Muhammed Ali Suleiman, Khloud Ahmed Saad Elsayed, Rahma AbdElfattah Ibrahim, Marwa M.I. Ghallab, Medical students’ health collaboration team, Ahmed F. Elbialy, Rafik F. Khalil, Mahmoud A. Said, Mariam Ashraf, Alaa A. Seleem, Sara A. Naeim, Mostafa Nemr, Rana A. Heigab, Ibrahim Ragheb, Mahmoud N. Mahmoud

**Affiliations:** 1 Faculty of Medicine, Kafr El-Shiekh University, Kafr El-Shiekh, Egypt; 2 Faculty of Medicine, Mansoura University, Mansoura, Egypt; 3 Department of Medical Parasitology, Faculty of Medicine, Kafrelsheikh University, Kafr El-Sheikh, Egypt

**Keywords:** Medical students, Stressors, Coping strategies, Integrated curriculum, Egypt, Academic stress

## Abstract

**Background:**

Egypt’s recent shift to an integrated (5+2) medical curriculum aims to enhance clinical competence but may also intensify stress among students. Understanding stressors and coping mechanisms is critical for promoting student well-being. This study aims to assess the levels and domains of stress, identify their predictors, and evaluate coping strategies among undergraduate medical students in Egypt.

**Methods:**

A multicenter cross-sectional study was conducted among 984 undergraduate medical students from Egyptian universities between April and May 2024 with response rate of 98.4%. Data were collected via a structured online questionnaire including demographics, the validated Medical Student Stressor Questionnaire (MSSQ), and the Coping Scale. Stressor domains were scored on a 0–4 Likert scale and coping on a 1–4 scale. Univariate and multivariate regression analyses identified predictors of stress and coping levels.Results:Academic-Related Stressors (ARS) recorded the highest mean score (2.97 ± 0.81), with “lack of time to review” and “large content load” as top stressors. Drive and Desire-Related Stressors (DRS) scored lowest (1.80 ± 1.18). High to severe stress prevalence was greatest in Academic Related Stress (ARS, 82.6%), followed by Group Activities-Related Stressors (GARS, 57.2%) and Interpersonal/Intrapersonal-Related Stressors (IRS, 54.7%). Female students showed significantly higher odds of stress in ARS, TLRS, SRS, DRS, and GARS and had lower coping scores (β = –0.21, p = 0.025). Rural residence increased odds of IRS (AOR = 1.67, p =0.047). Monthly income between 5,000 and 10,000 L.E. was associated with lower coping scores (β = –0.93, p = 0.040). Upcoming exams amplified stress across GARS,and TLRS. Chronic illness was linked to higher SRS (AOR = 1.81, p = 0.01). Overall, students relied more on emotion-focused and avoidance coping, while problem-focused strategies were less frequently employed.

**Conclusion:**

Egyptian medical students face substantial stress, predominantly academic in nature, under the integrated curriculum. Targeted institutional strategies—such as workload adjustments, clear academic expectations, anti-abuse measures, and resilience training—are needed to foster a healthier learning environment.

**Supplementary Information:**

The online version contains supplementary material available at 10.1186/s12909-025-08487-8.

## Introduction

Stress is a universal experience characterized by the physical and mental response to challenges or demands that disrupt the body’s balance [1, 2]. Stress is prevalent among university students due to a mix of academic pressures, social challenges, and the transition to adulthood. Research shows that the prevalence of stress in university students varies globally, ranging from 20% to 50%, with notable differences across different regions and fields of study [3].

University students face various stressors, including academic pressure, financial issues, and social conflicts. For medical students, these stressors are exacerbated by the intense and lengthy nature of their training [4]. Challenges they encounter consist of extensive study hours, demanding exams, clinical duties, and exposure to emotionally charged circumstances involving patients’ suffering and death. Such stressors can have significant implications, contributing to burnout, anxiety, depression, and diminished academic performance [5].

Focusing on medical students, especially, is important because they represent the healthcare system’s future. Chronic stress in this population impairs their well-being and can compromise their professional competence and quality of patient care [6]. In Egypt, the issue requires special attention because of limited resources, high expectations, and unique cultural pressures that amplify the challenges faced by medical students [6].

Coping mechanisms are strategies that manage stress and mitigate its impact. Among medical students, common mechanisms include problem-solving, seeking social support, engaging in recreational activities, and practicing mindfulness or relaxation techniques [2]. However, reliance on maladaptive coping strategies, such as avoidance or substance use, is not uncommon and may exacerbate stress-related outcomes [7] Understanding the coping mechanisms employed by Egyptian medical students can inform targeted interventions to enhance resilience and reduce the adverse effects of stress in this vulnerable group. Therefore, this study aimed to assess the types and levels of stressors, identify their predictors, and evaluate coping strategies among undergraduate medical students in Egypt.

## Methods

### Study design and settings

A multicenter cross-sectional study was conducted among undergraduate medical students in Egypt from April 1 to May 10, 2024. The study took place following the 2018 national medical education reform, which replaced the traditional curriculum with a five-year integrated program followed by two years of clinical training. By the time of data collection, all Egyptian medical schools had implemented the new system. The reform emphasized integration between basic and clinical sciences, shifting from traditional discipline-based teaching to a system-based, student-centered learning approach [8].

### Population

According to the Central Agency for Public Mobilization and Statistics (CAPMAS) for the academic year 2022/2023, the number of Egyptian medical students was approximately 73,000 [9]. At the time of data collection, there were more than 28 faculties of medicine across Egypt, most are public, as reported by the Ministry of Higher Education [10]. Participants aged 18 years or older who were enrolled as undergraduate medical students in Egyptian faculties of medicine and voluntarily consented to complete the online survey were included in the study.

### Data collection tool

Data were collected using a structured, web-based questionnaire in English. The tool consisted of four sections:

#### Introduction and consent

Provided an overview of the study, its objectives, and an informed consent statement

#### Demographics

Collected information on gender, residence, nationality, academic grade, university, family monthly income, history of chronic disease, and date of the next exam.

#### Medical student stressor questionnaire (MSSQ) [11, 12]

A validated and reliable 20-item tool rated on a 5-point Likert scale (0 = no stress to 4 = severe stress), assessing six domains: Academic-Related Stressors (ARS), Interpersonal and Intrapersonal-Related Stressors (IRS), Teaching and Learning-Related Stressors (TLRS), Social-Related Stressors (SRS), and Drive and Desire-Related Stressors (DRS).(11,12) – A validated and reliable 20-item tool rated on a 5-point Likert scale (0 = no stress to 4 = severe stress), assessing six domains: Academic-Related Stressors (ARS), Interpersonal and Intrapersonal-Related Stressors (IRS), Teaching and Learning-Related Stressors (TLRS), Social-Related Stressors (SRS), a

#### Coping scale [13]

A 13-item scale rated on a 4-point Likert scale (1 = not true about me to 4 = mostly true about me) measuring behavioral and appraisal coping strategies.

### Sampling and sample size

A convenience sampling method was used. The sample size was calculated using the *samplingbook* package in R [14]. For the 6-domain stressor scale (range: 0–4), the standard deviation was estimated as 1 using the range rule of thumb. Assuming a 95% confidence level, a 0.1 margin of error, and a standard deviation of 1, the minimum required sample was 385. Applying a design effect of 2, the target sample size was 770. In total, 984 students from different Egyptian universities completed the questionnaire ( supplementary Table 1).

### Data collection

Data was collected between April 1 and May 10, 2024. Investigators recruited collaborators from medical student communities across several Egyptian universities, which are Al-Azhar University, Alexandria University, Assiut University, Benha University, Cairo University, Mansoura University, Aswan University, and South Valley University. Collaborators distributed the survey link, created using Google Forms, through social media platforms including Facebook, Telegram, and WhatsApp, targeting eligible medical students.

### Statistical analysis

Analyses were conducted using R and Rstudio [15]. Data cleaning, analysis, and table generation were performed using the *tidyverse* and *gtsummary* packages. Categorical variables were summarized as frequencies and percentages, while continuous variables were presented as means and standard deviations.

Univariable and multivariable binary logistic regression models were applied to identify associated factors for each stressor domain. Similarly, univariable and multivariable linear regression models were used to identify associated factors of the coping score. For the linear regression models, standardized coefficients (β) were calculated by standardizing the coping score only, while independent variables were retained unstandardized. This approach yields coefficients representing the difference in outcome (in standard deviation units) between comparison groups.

Regression assumptions were assessed using diagnostic plots. Multicollinearity was evaluated using Generalized Variance Inflation Factors (GVIF). GVIF values for each model are presented in supplementary materials. All regression assumptions were satisfactorily met, and no substantial multicollinearity was detected.

Cluster-robust standard errors were used to calculate confidence intervals and p-values for all regression models, accounting for clustering of students within universities. Statistical significance was set at *p* < 0.05. The Benjamini-Hochberg correction was applied to all p-values from the multivariable models to control for multiple testing.

### Stress level categorization

Stress levels were classified according to the *Medical Student Stressor Questionnaire (MSSQ) Manual* [16]. According to the manual, mean domain scores of 0–1.00 indicate *mild* stress, 1.01–2.00 *moderate* stress, 2.01–3.00 *high* stress, and 3.01–4.00 *severe* stress.In our analysis, *high* and *severe* levels (indicating stress affecting daily activities) were combined into one group, while *mild* and *moderate* levels were combined into another. This categorization has been used to allow for more interpretable regression analysis.

## Results

Of 1,067 responses, 984 were included in the final analysis after removing inconsistent data. Participant characteristics are summarized in Table 1. The sample comprised 58% males (*n* = 571) and 42% females (*n* = 413). Most respondents resided in urban areas (62.8%, *n* = 618), were in the 5th academic year (41.4%, *n* = 407), reported a monthly household income of < 5,000 L.E. (68.9%, *n* = 678), and had their next exam scheduled in one month or more (46.2%, *n* = 455).

**Table 1:** Characteristics of the study sample of medical students in Egypt (*N* = 984)

Variable	*n* (%)
Sex	
Male	571 (58.0%)
Female	413 (42.0%)
**Residence**	
Urban	618 (62.8%)
Rural	366 (37.2%)
**Grade**	
1st	103 (10.5%)
2nd	81 (8.2%)
3rd	163 (16.6%)
4th	230 (23.4%)
5th	407 (41.4%)
**Monthly income**	
Less than 5000 L.E	678 (68.9%)
5000–10,000 L.E	201 (20.4%)
More than 10,000 L.E	105 (10.7%)
**Chronic disease**	
No	893 (90.8%)
Yes	91 (9.2%)
**Next exam**	
After more than month	423 (43.0%)
After one week but less than month	455 (46.2%)
This week	106 (10.8%)

As shown in Table 2, the highest mean (SD) stressor score was recorded for Academic-Related Stressors (ARS) at 2.97 (± 0.81; 95%CI: 2.92, 3.02 ). Within this domain, the most stressful items were *“Lack of time to review what has been learned”* (3.10 ± 1.02; 95%CI:3.04, 3.1 ) and *“A large amount of content to be learned”* (3.09 ± 1.02; 95%CI: 3.02, 3.15). The lowest mean score was for Drive and Desire-Related Stressors (DRS) at 1.80 (± 1.18; 95%CI:1.73, 1.87 ), with the least stressful items being *“Parental wish for you to study medicine”* (1.78 ± 1.37; 95%CI:1.70, 1.87) and *“Unwillingness to study medicine”* (1.82 ± 1.34; 95%CI:1.73, 1.9).

**Table 2:** Sources of stress among medical students in Egypt (*N* = 984)

Stressor domain	Items	Mean (SD)	95% CI	Range
**ARS**	Tests/examinations	2.94 (1.08)	2.87, 3.01	0–4
	Falling behind in reading schedule	2.81 (1.11)	2.74, 2.88	0–4
	Large amount of content to be learnt	3.09 (1.02)	3.02, 3.15	0–4
	Lack of time to review what have been learnt	3.10 (1.02)	3.04, 3.17	0–4
	Heavy workload	2.90 (0.98)	2.83, 2.96	0–4
	**Total ARS**	**2.97 (0.81)**	**2.92**,** 3.02**	**0–4**
**IRS**	Verbal or physical abuse by other student(s)	2.08 (1.23)	2.01, 2.16	0–4
	Verbal or physical abuse by teacher(s)	2.36 (1.22)	2.29, 2.44	0–4
	Verbal or physical abuse by personnel(s)	2.17 (1.20)	2.09, 2.24	0–4
	Conflict with teacher(s)	2.26 (1.18)	2.19, 2.33	0–4
	**Total IRS**	**2.22 (1.04)**	**2.15**,** 2.28**	**0–4**
**TLRS**	Not enough feedback from teacher (s)	1.90 (1.18)	1.82, 1.97	0–4
	Uncertainty of what is expected of me	2.35 (1.06)	2.29, 2.42	0–4
	Lack of recognition for work done	2.24 (1.06)	2.18, 2.31	0–4
	**Total TLRS**	**2.16 (0.87)**	**2.11**,** 2.22**	**0–4**
**SRS**	Unable to answer questions from patients	2.26 (1.10)	2.19, 2.33	0–4
	Talking to patients about personal problems	1.84 (1.12)	1.77, 1.91	0–4
	Facing illness or death of the patients	2.42 (1.15)	2.35, 2.49	0–4
	**Total SRS**	**2.17 (0.86)**	**2.12**,** 2.23**	**0–4**
**DRS**	Unwillingness to study medicine	1.82 (1.34)	1.73, 1.90	0–4
	Parental wish for you to study medicine	1.78 (1.37)	1.70, 1.87	0–4
	**Total DRS**	**1.80 (1.18)**	**1.73**,** 1.87**	**0–4**
**GARS**	Participation in class presentation	2.13 (1.16)	2.06, 2.20	0–4
	Need to do well (imposed by others)	2.36 (1.13)	2.29, 2.43	0–4
	Feeling of incompetence	2.48 (1.11)	2.41, 2.55	0–4
	**Total GARS**	**2.33 (0.88)**	**2.27**,** 2.38**	**0–4**

*Abbreviations*: *ARS *Academic Related Stressors, *IRS *Interpersonal and Intrapersonal Related Stressors, *TLRS *Teaching and Learning Related Stressors, *SRS *Social Related Stressors, *DRS *Drive and Desire Related Stressors, *GARS *Group Activities Related Stressors

**Fig. 1:**
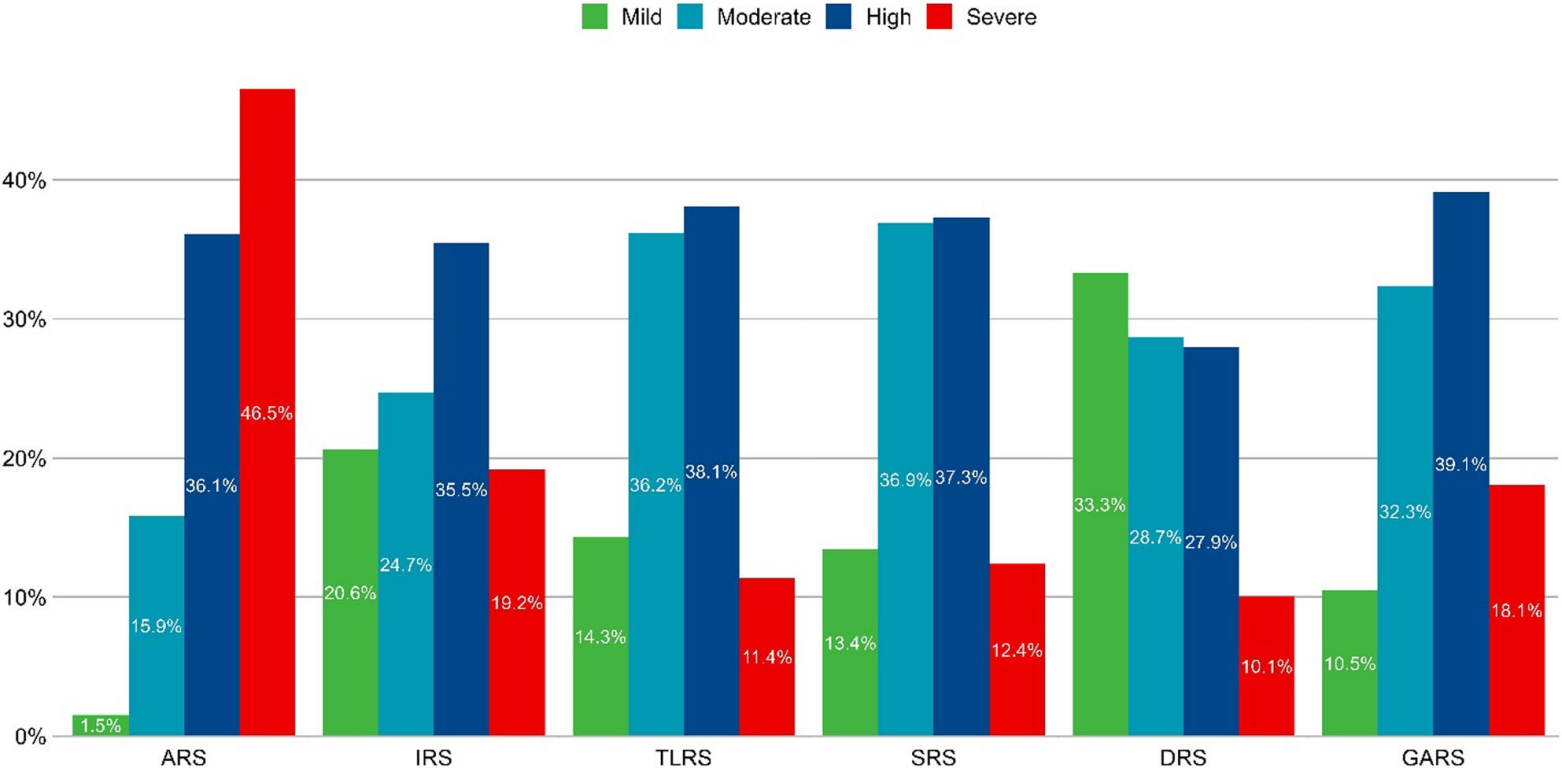
Stress levels across the six domains in medical students

Figure 1 illustrates the prevalence and severity of stress across different stressor domains. The proportions of students experiencing high and severe stress were as follows: ARS (36.1%, 46.5%), IRS (35.5%, 19.2%), TLRS (38.1%, 11.4%), SRS (37.3%, 12.4%), DRS (27.9%, 10.1%), and GARS (39.1%, 18.1%).

### Regression analysis of demographic predictors of stressors

After adjusting for potential confounders (Tables 3, 4, 5, 6, 7 and 8), female students demonstrated significantly higher odds of experiencing stress in multiple domains, including ARS (AOR = 2.15, *p* < 0.001), SRS (AOR = 1.69, *p* = 0.007), DRS (AOR = 1.48, *p* = 0.003), and GARS (OR = 1.72, *p* = 0.004). Students from rural areas had higher odds of stress in IRS (AOR = 1.67, *p* = 0.047). Regarding academic year, third-year students showed elevated odds in DRS (AOR = 1.55, *p* = 0.025) and GARS(AOR = 2.15, *p* = 0.033), fourth-year students in IRS, TLRS (AOR = 2.28, *p* = 0.019), SRS(AOR = 1.96, *p* = 0.032), DRS(AOR = 2.88, *p* < 0.001), and GARS(AOR = 3.13, *p* = 0.017). The presence of chronic illness was associated with increased odds in SRS (AOR = 1.81, *p* = 0.010). A monthly family income between 5,000 and 10,000 L.E. was linked to lower odds in IRS (AOR = 0.64, *p* = 0.045). Exam timing also influenced stress levels: having exams in more than one week but less than one month was associated with higher odds in TLRS(AOR = 2.01, *p* = 0.035), and GARS(AOR = 2.52, *p* = 0.02).

**Table 3:** Binary logistic regression analysis of having ARS among medical students in Egypt (*N* = 984)

Variable	ARS		Univariable regression		Multivariable regression		
	No *N* = 171^1,2^	Yes *N* = 813^1,2,3^	COR (95% CI)	*p*-value	AOR (95% CI)	*p*-value	q-value^4^
**Sex**							
Male	122 (21.4%)	449 (78.6%)	Reference		Reference		
Female	49 (11.9%)	364 (88.1%)	2.02 (1.17, 3.49)	**0.012**	2.15 (1.38, 3.35)	**< 0.001**	**0.035**
**Residence**							
Urban	91 (14.7%)	527 (85.3%)	Reference		Reference		
Rural	80 (21.9%)	286 (78.1%)	0.62 (0.38, 1.01)	0.054	0.73 (0.55, 0.97)	**0.032**	0.18
**Grade**							
1st	27 (26.2%)	76 (73.8%)	Reference		Reference		
2nd	22 (27.2%)	59 (72.8%)	0.95 (0.28, 3.21)	0.94	1.02 (0.31, 3.40)	0.97	0.98
3rd	35 (21.5%)	128 (78.5%)	1.30 (0.44, 3.83)	0.63	1.41 (0.48, 4.16)	0.53	0.72
4th	50 (21.7%)	180 (78.3%)	1.28 (0.32, 5.14)	0.73	1.51 (0.41, 5.56)	0.54	0.72
5th	37 (9.1%)	370 (90.9%)	3.55 (0.71, 17.66)	0.12	3.26 (0.76, 13.90)	0.11	0.31
**Monthly income**							
Less than 5000 L.E	114 (16.8%)	564 (83.2%)	Reference		Reference		
5000–10,000 L.E	48 (23.9%)	153 (76.1%)	0.64 (0.36, 1.14)	0.13	0.64 (0.41, 0.99)	**0.045**	0.21
More than 10,000 L.E	9 (8.6%)	96 (91.4%)	2.16 (0.86, 5.39)	0.10	1.93 (0.87, 4.31)	0.11	0.31
**Chronic disease**							
No	156 (17.5%)	737 (82.5%)	Reference		Reference		
Yes	15 (16.5%)	76 (83.5%)	1.07 (0.62, 1.85)	0.80	1.01 (0.60, 1.72)	0.96	0.98
**Next exam**							
After more than month	58 (13.7%)	365 (86.3%)	Reference		Reference		
After one week but less than month	87 (19.1%)	368 (80.9%)	0.67 (0.30, 1.48)	0.33	0.80 (0.52, 1.26)	0.34	0.55
This week	26 (24.5%)	80 (75.5%)	0.49 (0.20, 1.21)	0.12	0.65 (0.38, 1.08)	0.10	0.31

*Abbreviations*: *AOR *Adjusted Odds Ratio, *ARS *Academic Related Stressors, *CI *Confidence Interval, *COR *Crude Odds Ratio

^1^n (%)

^2^No = Mild and Moderate, Yes = High and Severe

^3^Prevalence of High/Severe ARS = 82.6% (95% CI = 80.1%, 84.9%)

^4^Benjamini & Hochberg correction for multiple testing

**Table 4:** Binary logistic regression analysis of having IRS among medical students in Egypt (*N* = 984)

Variable	IRS		Univariable regression		Multivariable regression		
	No *N* = 446^1,2^	Yes *N* = 538^1,2,3^	COR (95% CI)	*p*-value	AOR (95% CI)	*p*-value	q-value^4^
**Sex**							
Male	263 (46.1%)	308 (53.9%)	Reference		Reference		
Female	183 (44.3%)	230 (55.7%)	1.07 (0.62, 1.87)	0.80	1.22 (0.81, 1.84)	0.34	0.55
**Residence**							
Urban	316 (51.1%)	302 (48.9%)	Reference		Reference		
Rural	130 (35.5%)	236 (64.5%)	1.90 (0.91, 3.97)	0.088	1.67 (1.01, 2.76)	**0.047**	0.21
**Grade**							
1st	50 (48.5%)	53 (51.5%)	Reference		Reference		
2nd	32 (39.5%)	49 (60.5%)	1.44 (0.76, 2.75)	0.26	1.64 (0.85, 3.15)	0.14	0.33
3rd	63 (38.7%)	100 (61.3%)	1.50 (0.84, 2.66)	0.17	1.70 (0.88, 3.30)	0.12	0.32
4th	72 (31.3%)	158 (68.7%)	2.07 (0.90, 4.77)	0.087	2.57 (0.94, 7.03)	0.067	0.23
5th	229 (56.3%)	178 (43.7%)	0.73 (0.23, 2.38)	0.61	0.93 (0.44, 1.98)	0.86	0.96
**Monthly income**							
Less than 5000 L.E	303 (44.7%)	375 (55.3%)	Reference		Reference		
5000–10,000 L.E	93 (46.3%)	108 (53.7%)	0.94 (0.36, 2.43)	0.90	0.85 (0.49, 1.48)	0.56	0.73
More than 10,000 L.E	50 (47.6%)	55 (52.4%)	0.89 (0.32, 2.50)	0.82	0.89 (0.45, 1.75)	0.73	0.85
**Chronic disease**							
No	412 (46.1%)	481 (53.9%)	Reference		Reference		
Yes	34 (37.4%)	57 (62.6%)	1.44 (0.69, 2.97)	0.33	1.56 (0.87, 2.81)	0.14	0.33
**Next exam**							
After more than month	215 (50.8%)	208 (49.2%)	Reference		Reference		
After one week but less than month	184 (40.4%)	271 (59.6%)	1.52 (0.53, 4.40)	0.44	1.53 (0.74, 3.15)	0.25	0.46
This week	47 (44.3%)	59 (55.7%)	1.30 (0.39, 4.30)	0.67	1.05 (0.48, 2.27)	0.91	0.98

*Abbreviations*: *AOR *Adjusted Odds Ratio, *CI *Confidence Interval, *COR *Crude Odds Ratio, *IRS *Interpersonal and Intrapersonal Related Stressors

^1^n (%)

^2^No = Mild and Moderate, Yes = High and Severe

^3^Prevalence of High/Severe IRS = 54.7% (95% CI = 51.6%, 57.8%)

^4^Benjamini & Hochberg correction for multiple testing

**Table 5:** Binary logistic regression analysis of having TLRS among medical students in Egypt (*N* = 984)

Variable	TLRS		Univariable regression		Multivariable regression		
	No *N* = 497^1,2^	Yes *N* = 487^1,2,3^	COR (95% CI)	*p*-value	AOR (95% CI)	*p*-value	q-value^4^
**Sex**							
Male	305 (53.4%)	266 (46.6%)	Reference		Reference		
Female	192 (46.5%)	221 (53.5%)	1.32 (0.84, 2.07)	0.23	1.44 (0.99, 2.09)	0.054	0.23
**Residence**							
Urban	340 (55.0%)	278 (45.0%)	Reference		Reference		
Rural	157 (42.9%)	209 (57.1%)	1.63 (0.80, 3.30)	0.18	1.40 (0.87, 2.26)	0.16	0.36
**Grade**							
1st	50 (48.5%)	53 (51.5%)	Reference		Reference		
2nd	37 (45.7%)	44 (54.3%)	1.12 (0.69, 1.81)	0.64	1.36 (0.75, 2.48)	0.31	0.54
3rd	69 (42.3%)	94 (57.7%)	1.29 (0.80, 2.07)	0.30	1.54 (0.88, 2.71)	0.13	0.33
4th	89 (38.7%)	141 (61.3%)	1.49 (0.97, 2.31)	0.070	2.28 (1.14, 4.55)	**0.019**	0.17
5th	252 (61.9%)	155 (38.1%)	0.58 (0.21, 1.57)	0.28	0.82 (0.46, 1.46)	0.50	0.71
**Monthly income**							
Less than 5000 L.E	354 (52.2%)	324 (47.8%)	Reference		Reference		
5000–10,000 L.E	91 (45.3%)	110 (54.7%)	1.32 (0.60, 2.93)	0.49	1.14 (0.64, 2.04)	0.65	0.78
More than 10,000 L.E	52 (49.5%)	53 (50.5%)	1.11 (0.45, 2.74)	0.81	1.02 (0.53, 1.97)	0.94	0.98
**Chronic disease**							
No	457 (51.2%)	436 (48.8%)	Reference		Reference		
Yes	40 (44.0%)	51 (56.0%)	1.34 (0.65, 2.73)	0.43	1.39 (0.75, 2.57)	0.29	0.51
**Next exam**							
After more than month	254 (60.0%)	169 (40.0%)	Reference		Reference		
After one week but less than month	190 (41.8%)	265 (58.2%)	2.10 (0.85, 5.16)	0.11	2.01 (1.05, 3.86)	**0.035**	0.18
This week	53 (50.0%)	53 (50.0%)	1.50 (0.60, 3.75)	0.38	1.17 (0.65, 2.10)	0.60	0.76

*Abbreviations*: *AOR *Adjusted Odds Ratio, *CI *Confidence Interval, *COR *Crude Odds Ratio, *TLRS *Teaching and Learning Related Stressors

^1^n (%)

^2^No = Mild and Moderate, Yes = High and Severe

^3^Prevalence of High/Severe TLRS = 49.5% (95% CI = 46.4%, 52.6%)

^4^Benjamini & Hochberg correction for multiple testing

**Table 6:** Binary logistic regression analysis of having SRS among medical students in Egypt (*N* = 984)

Variable	SRS		Univariable regression		Multivariable regression		
	No *N* = 495^1,2^	Yes *N* = 489^1,2,3^	COR (95% CI)	*p*-value	AOR (95% CI)	*p*-value	q-value^4^
**Sex**							
Male	312 (54.6%)	259 (45.4%)	Reference		Reference		
Female	183 (44.3%)	230 (55.7%)	1.51 (1.02, 2.24)	**0.038**	1.69 (1.15, 2.49)	**0.007**	0.11
**Residence**							
Urban	332 (53.7%)	286 (46.3%)	Reference		Reference		
Rural	163 (44.5%)	203 (55.5%)	1.45 (0.77, 2.72)	0.25	1.20 (0.86, 1.67)	0.28	0.51
**Grade**							
1st	48 (46.6%)	55 (53.4%)	Reference		Reference		
2nd	30 (37.0%)	51 (63.0%)	1.48 (0.75, 2.92)	0.25	1.76 (0.96, 3.22)	0.067	0.23
3rd	69 (42.3%)	94 (57.7%)	1.19 (0.71, 2.00)	0.51	1.42 (0.82, 2.46)	0.22	0.44
4th	93 (40.4%)	137 (59.6%)	1.29 (0.79, 2.09)	0.31	1.96 (1.06, 3.63)	**0.032**	0.18
5th	255 (62.7%)	152 (37.3%)	0.52 (0.16, 1.68)	0.28	0.71 (0.33, 1.53)	0.38	0.60
**Monthly income**							
Less than 5000 L.E	347 (51.2%)	331 (48.8%)	Reference		Reference		
5000–10,000 L.E	93 (46.3%)	108 (53.7%)	1.22 (0.57, 2.60)	0.61	0.96 (0.62, 1.49)	0.87	0.96
More than 10,000 L.E	55 (52.4%)	50 (47.6%)	0.95 (0.37, 2.48)	0.92	0.78 (0.34, 1.76)	0.54	0.72
**Chronic disease**							
No	460 (51.5%)	433 (48.5%)	Reference		Reference		
Yes	35 (38.5%)	56 (61.5%)	1.70 (0.99, 2.92)	0.054	1.81 (1.15, 2.84)	**0.010**	0.13
**Next exam**							
After more than month	256 (60.5%)	167 (39.5%)	Reference		Reference		
After one week but less than month	193 (42.4%)	262 (57.6%)	2.08 (0.78, 5.58)	0.15	1.99 (0.98, 4.03)	0.057	0.23
This week	46 (43.4%)	60 (56.6%)	2.00 (0.74, 5.42)	0.17	1.54 (0.73, 3.23)	0.25	0.46

*Abbreviations*: *AOR *Adjusted Odds Ratio, *CI *Confidence Interval, *COR *Crude Odds Ratio, *SRS *Social Related Stressors

^1^n (%)

^2^No = Mild and Moderate, Yes = High and Severe

^3^Prevalence of High/Severe SRS = 49.7% (95% CI = 46.6%, 52.8%)

^4^Benjamini & Hochberg correction for multiple testing

**Table 7:** Binary logistic regression analysis of having DRS among medical students in Egypt (*N* = 984)

Variable	DRS		Univariable regression		Multivariable regression		
	No N = 610^1,2^	Yes N = 374^1,2,3^	COR (95% CI)	p-value	AOR(95% CI)	p-value	q-value^4^
Sex							
Male	370 (64.8%)	201 (35.2%)	Reference		Reference		
Female	240 (58.1%)	173 (41.9%)	1.33 (0.98, 1.79)	0.063	1.48 (1.14, 1.91)	0.003	0.081
Residence							
Urban	401 (64.9%)	217 (35.1%)	Reference		Reference		
Rural	209 (57.1%)	157 (42.9%)	1.39 (0.80, 2.41)	0.24	1.25 (0.86, 1.83)	0.25	0.46
Grade							
1st	69 (67.0%)	34 (33.0%)	Reference		Reference		
2nd	51 (63.0%)	30 (37.0%)	1.19 (0.71, 2.00)	0.50	1.37 (0.86, 2.19)	0.18	0.38
3rd	98 (60.1%)	65 (39.9%)	1.35 (0.92, 1.96)	0.12	1.55 (1.06, 2.28)	0.025	0.18
4th	116 (50.4%)	114 (49.6%)	1.99 (1.34, 2.97)	<0.001	2.88 (1.54, 5.37)	<0.001	0.035
5th	276 (67.8%)	131 (32.2%)	0.96 (0.40, 2.34)	0.93	1.33 (0.89, 1.97)	0.16	0.36
Monthly income							
Less than 5000 L.E	425 (62.7%)	253 (37.3%)	Reference		Reference		
5000 - 10000 L.E	121 (60.2%)	80 (39.8%)	1.11 (0.56, 2.19)	0.76	1.02 (0.64, 1.61)	0.94	0.98
More than 10000 L.E	64 (61.0%)	41 (39.0%)	1.08 (0.60, 1.93)	0.81	1.02 (0.66, 1.58)	0.93	0.98
Chronic disease							
No	556 (62.3%)	337 (37.7%)	Reference		Reference		
Yes	54 (59.3%)	37 (40.7%)	1.13 (0.71, 1.79)	0.60	1.17 (0.79, 1.74)	0.44	0.67
Next exam							
After more than month	293 (69.3%)	130 (30.7%)	Reference		Reference		
After one week but less than month	258 (56.7%)	197 (43.3%)	1.72 (0.74, 4.00)	0.21	1.82 (0.96, 3.46)	0.066	0.23
This week	59 (55.7%)	47 (44.3%)	1.80 (0.64, 5.01)	0.26	1.68 (0.83, 3.38)	0.15	0.34

*Abbreviations*: *AOR *Adjusted Odds Ratio, *CI *Confidence Interval, *COR *Crude Odds Ratio, *DRS *Drive and Desire Related Stressors

^1^n (%)

^2^No = Mild and Moderate, Yes = High and Severe

^3^Prevalence of High/Severe DRS = 38.0% (95% CI = 35.0%, 41.1%)

^4^Benjamini & Hochberg correction for multiple testing

**Table 8:** Binary logistic regression analysis of having GARS among medical students in Egypt (*N* = 984)

Variable	GARS		Univariable regression		Multivariable regression		
	No *N* = 421^1,2^	Yes *N* = 563^1,2,3^	COR (95% CI)	*p*-value	AOR (95% CI)	*p*-value	q-value^4^
**Sex**							
Male	270 (47.3%)	301 (52.7%)	Reference		Reference		
Female	151 (36.6%)	262 (63.4%)	1.56 (1.04, 2.32)	**0.030**	1.72 (1.19, 2.50)	**0.004**	0.081
**Residence**							
Urban	277 (44.8%)	341 (55.2%)	Reference		Reference		
Rural	144 (39.3%)	222 (60.7%)	1.25 (0.59, 2.66)	0.56	1.08 (0.68, 1.70)	0.76	0.86
**Grade**							
1st	48 (46.6%)	55 (53.4%)	Reference		Reference		
2nd	32 (39.5%)	49 (60.5%)	1.34 (0.60, 2.97)	0.48	1.73 (0.84, 3.56)	0.13	0.33
3rd	56 (34.4%)	107 (65.6%)	1.67 (0.71, 3.92)	0.24	2.15 (1.07, 4.36)	**0.033**	0.18
4th	76 (33.0%)	154 (67.0%)	1.77 (0.76, 4.09)	0.18	3.13 (1.23, 7.94)	**0.017**	0.17
5th	209 (51.4%)	198 (48.6%)	0.83 (0.20, 3.41)	0.79	1.27 (0.52, 3.14)	0.60	0.76
**Monthly income**							
Less than 5000 L.E	303 (44.7%)	375 (55.3%)	Reference		Reference		
5000–10,000 L.E	75 (37.3%)	126 (62.7%)	1.36 (0.59, 3.15)	0.48	1.13 (0.68, 1.88)	0.63	0.77
More than 10,000 L.E	43 (41.0%)	62 (59.0%)	1.17 (0.53, 2.56)	0.70	1.01 (0.53, 1.93)	0.98	0.98
**Chronic disease**							
No	380 (42.6%)	513 (57.4%)	Reference		Reference		
Yes	41 (45.1%)	50 (54.9%)	0.90 (0.48, 1.71)	0.76	0.89 (0.53, 1.48)	0.65	0.78
**Next exam**							
After more than month	230 (54.4%)	193 (45.6%)	Reference		Reference		
After one week but less than month	149 (32.7%)	306 (67.3%)	2.45 (0.88, 6.84)	0.088	2.52 (1.16, 5.47)	**0.020**	0.17
This week	42 (39.6%)	64 (60.4%)	1.82 (0.67, 4.90)	0.24	1.57 (0.82, 2.99)	0.17	0.37

*Abbreviations*: *AOR* Adjusted Odds Ratio, *CI* Confidence Interval, *COR* Crude Odds Ratio, *GARS* Group Activities Related Stressors

^1^n (%)

^2^No = Mild and Moderate, Yes = High and Severe

^3^Prevalence of High/Severe GARS = 57.2% (95% CI = 54.1%, 60.3%)

^4^Benjamini & Hochberg correction for multiple testing

### Coping score analysis

Linear regression analysis (Table 9) revealed that females had significantly lower coping scores compared to males (β = – 0.21, *p* = 0.025 ).

**Table 9:** Linear regression analysis of coping scores among medical students in Egypt (*N* = 984)

Variable	Coping Score^1^	Univariable regression			Multivariable regression			
		B (95% CI)	β (95% CI)	*p*-value	B (95% CI)	β (95% CI)	*p*-value	q-value^2^
**Overall**	34.5 (5.6)							
**Sex**								
Male	35.1 (5.2)	Reference	Reference		Reference	Reference		
Female	33.8 (6.0)	-1.25 (-2.40, -0.09)	-0.22 (-0.43, -0.02)	**0.034**	-1.16 (-2.17, -0.15)	-0.21 (-0.39, -0.03)	**0.025**	0.18
**Residence**								
Urban	34.8 (5.5)	Reference	Reference		Reference	Reference		
Rural	34.1 (5.6)	-0.67 (-2.43, 1.09)	-0.12 (-0.44, 0.20)	0.45	-0.38 (-1.30, 0.54)	-0.07 (-0.23, 0.10)	0.42	0.64
**Grade**								
1st	33.7 (5.8)	Reference	Reference		Reference	Reference		
2nd	33.6 (5.7)	-0.14 (-1.58, 1.29)	-0.03 (-0.28, 0.23)	0.85	-0.38 (-1.95, 1.19)	-0.07 (-0.35, 0.21)	0.63	0.77
3rd	34.4 (5.3)	0.63 (-0.69, 1.95)	0.11 (-0.12, 0.35)	0.35	0.40 (-0.81, 1.62)	0.07 (-0.15, 0.29)	0.52	0.72
4th	34.1 (5.0)	0.36 (-0.81, 1.52)	0.06 (-0.15, 0.27)	0.55	-0.53 (-2.04, 0.98)	-0.10 (-0.37, 0.18)	0.49	0.71
5th	35.2 (5.8)	1.48 (-1.22, 4.18)	0.27 (-0.22, 0.75)	0.28	0.75 (-0.84, 2.33)	0.13 (-0.15, 0.42)	0.36	0.57
**Monthly income**								
Less than 5000 L.E	34.9 (5.3)	Reference	Reference		Reference	Reference		
5000–10,000 L.E	33.6 (6.3)	-1.30 (-3.05, 0.45)	-0.23 (-0.55, 0.08)	0.15	-0.93 (-2.04, 0.18)	-0.17 (-0.37, 0.03)	0.10	0.31
More than 10,000 L.E	34.1 (5.2)	-0.82 (-2.62, 0.98)	-0.15 (-0.47, 0.18)	0.37	-0.51 (-1.84, 0.82)	-0.09 (-0.33, 0.15)	0.45	0.67
**Chronic disease**								
No	34.5 (5.5)	Reference	Reference		Reference	Reference		
Yes	34.6 (5.8)	0.12 (-1.99, 2.24)	0.02 (-0.36, 0.40)	0.91	0.29 (-1.57, 2.14)	0.05 (-0.28, 0.39)	0.76	0.86
**Next exam**								
After more than month	35.4 (5.3)	Reference	Reference		Reference	Reference		
After one week but less than month	34.0 (5.5)	-1.32 (-3.62, 0.99)	-0.24 (-0.65, 0.18)	0.26	-1.01 (-2.62, 0.61)	-0.18 (-0.47, 0.11)	0.22	0.44
This week	33.4 (6.4)	-1.97 (-4.68, 0.74)	-0.35 (-0.84, 0.13)	0.15	-1.43 (-3.14, 0.29)	-0.26 (-0.57, 0.05)	0.10	0.31

*Abbreviations*: *B *Unstandardized Coefficient, *CI *Confidence Interval, *β *Standardized Coefficient (outcome standardized, independent variables unstandardized)

^1^Mean (SD)

^2^Benjamini & Hochberg correction for multiple testing

## Discussion

The new integration system (5 + 2) adopted by medical faculties in Egypt represents a major reform in undergraduate medical education [8, 17]. Under this system, students undergo five years of academic education integrating both basic and clinical sciences from the early stages, followed by two years of clinical internship (house officer training). This competency-based curriculum replaces the old 6 + 1 model and aims to enhance early clinical exposure, improve practical skills, and align medical training with international standards [8, 17]. The early exposure to clinical settings, continuous assessments, and high academic workload could intensify Academic-Related Stressors (ARS) and Teaching and Learning-Related Stressors (TLRS). Additionally, adapting to the demands of teamwork and new learning styles may heighten Group Activities-Related Stressors (GARS) and Intrapersonal and Interpersonal-Related Stressors (IRS). At the same time, changes in social dynamics and support systems could further contribute to Social-Related Stressors (SRS) [17, 18]. Hence, our study aims to assess the stressors and coping strategies among medical students in Egypt after reforming the medical education into the integrated system.

Our study found that the highest mean score was in the Academic-Related Stressors (ARS) domain (mean = 2.97), indicating a high level of stress approaching the severe range. This suggests that academic demands constitute a major source of psychological burden among the participants. The mismatch between students’ perceived capacities and the curriculum’s cognitive and temporal demands reflects a person–environment misfit contributing to stress [19].

Most students (82.6%) experienced high to severe stress levels related to academic demands. This is consistent with previous studies reporting the highest scores from ARS that caused high stress levels ( [18, 20–23]. The severe stress reported was from the lack of time for revision and the large amount of content, with mean scores of 3.1 and 3.09, respectively, in our study. which aligns with cognitive-load theory, as continuous assessments and large content volumes amplify mental workload, and with self-determination theory, which links constrained autonomy to reduced coping efficacy [24]. The integrated medical curriculum is characterized by high intrinsic load due to complex and dense content, while frequent assessments, tight schedules, and ambiguous expectations contribute to extraneous load. When these demands exceed students’ working-memory capacity, cognitive overload occurs, resulting in heightened stress and reduced learning efficiency. The elevated ARS scores observed in our sample are therefore consistent with excessive intrinsic and extraneous cognitive load imposed by the curricular structure.

The perceived shortage of time for adequate revision may stem from tightly packed academic schedules, frequent assessments, or competing responsibilities, all of which limit students’ ability to thoroughly review and consolidate their learning [8]. Similarly, the stress associated with a large volume of content reflects the cognitive burden placed on students to process and retain extensive material within limited timeframes.

These stressors can negatively affect academic performance and contribute to mental fatigue and anxiety [25, 26]. The prominence of high and sever forms of ARS suggests a need for academic institutions to reconsider workload distribution, pacing of content delivery, and opportunities for revision, to support students’ academic success and psychological well-being.

When assessing interpersonal and intrapersonal-related stressors (IRS) among medical students, our study reported an overall mean score of 2.22, with 54.7% of students experiencing high to severe IRS. This finding is supported by previous studies, which reported similar levels among medical students—94.5% at Zagazig University in Egypt, 39.1% at King Khalid University in Saudi Arabia, and 25.5% at Helwan University [18, 21, 23]. In our study, the highest mean scores for IRS types were found in verbal and physical abuse by teachers (mean score: 2.36), followed closely by conflicts with teachers (mean score: 2.26), reflecting high stress.

Previous studies have consistently shown that medical students experience various forms of abuse and mistreatment from professors, colleagues, and healthcare staff, which is linked to increased stress and impaired academic performance. Research from the 1990s indicated that 33% of students faced verbal abuse from resident doctors, while more recent data from Egypt and Lebanon highlighted high levels of mistreatment, including verbal, physical, and social exclusion, with fellow students contributing significantly to the issue [18, 21, 23, 27, 28]. A global systematic review further emphasized the widespread nature of this problem, documenting abuse by peers, seniors, and professors in medical schools, particularly in the United States [29].

The abuse—whether verbal, physical, or behavioral—not only undermines student morale and mental health but also jeopardizes their academic progress, professional identity formation, and future clinical competence [27]. These findings warrant immediate attention and institutional reform. Addressing this issue requires a multifaceted approach, including establishing clear anti-abuse policies, confidential reporting systems, faculty training in respectful communication and conflict resolution, and integrating wellness and psychological support services for students.

The findings on teaching and learning-related stressors (TLRS), with an overall mean score of 2.16, reflect a substantial level of stress among medical students, particularly as 49.5% reported high to severe levels of stress.

This pattern is consistent with prior studies, such as those conducted at King Khalid University, Nigeria, and Helwan University, all of which reported similarly high levels of TLRS stress [18, 21, 22]. The findings underscore the persistent pressure medical students face across diverse settings due to the rigorous nature of medical education.

The highest-rated item in this domain—uncertainty about what is expected of students (mean score: 2.35)—indicates that unclear academic expectations are a key stressor. This ambiguity can lead to confusion, anxiety, and reduced academic confidence, particularly in highly structured and performance-driven environments [30]. Addressing this issue through clearer communication, transparent assessment criteria, and consistent academic guidance could significantly reduce TLRS and improve the educational experience for medical students. According to Self-Determination Theory, unmet needs for autonomy, competence, and relatedness under the integrated system may heighten stress and weaken coping. This theory proposes that psychological well-being and effective self-regulation depend on the fulfillment of three basic needs: autonomy, competence, and relatedness. Stress arises when these needs are hindered. In our study, unclear academic expectations, inconsistent feedback, and limited time may undermine competence; rigid curricular demands may restrict autonomy; and interpersonal stressors may weaken a sense of relatedness. These unmet needs help explain the elevated stress levels, gender differences, and reliance on emotion-focused coping observed among our participants [31].

In our study, the mean score for social-related stressors (SRS) was 2.17, with 49.7% of participants reporting high to severe levels of stress. This finding aligns with previous research, which has consistently reported similar rates of significant social-related stress among students [18, 21, 23]. The highest-rated item in this domain was the stress caused by facing the illness or death of patients, as well as the inability to answer questions from patients.

The findings suggest that emotional and ethical dilemmas, particularly those involving patient care and the pressure to provide accurate, compassionate responses, contribute significantly to social-related stress. These challenges reflect the complexity of medical education, where students are required to manage not only academic demands but also emotionally taxing situations in their interactions with patients. The need for emotional resilience training and support systems in medical curricula is critical to address these stressors effectively.

Regarding Drive and Desire stressors (DRS), this domain recorded the lowest mean score (1.8), indicating a moderate stressor. The key stress-inducing items were “unwillingness to study medicine” and “parental wish to pursue medicine.”

This reflects internal conflicts and a lack of personal motivation as underlying stressors. Nearly 62% of students reported mild to moderate levels of stress as DRS. This finding is consistent with previous studies that also reported predominantly mild to moderate stress levels in this area [18, 21]. However, it is important to note that the proportion of students experiencing high to severe stress (38%), though smaller, was not negligible. The results highlight the ongoing need to address motivational struggles and external pressures in medical education, particularly by offering career guidance and psychological support to students who may be misaligned with their chosen field.

Regarding group activities-related stressors (GRAS) among medical students, the mean score was 2.33, reflecting a high stressor. The most prominent stressor was the feeling of incompetence and the pressure to perform well as imposed by others. This highlights the intense expectations often experienced in collaborative academic settings. About 57.2% of students reported high to severe levels of stress related to GRAS, indicating that group work is a substantial source of pressure in medical education. This underscores the need to reassess how group work is structured and supported in medical education to reduce its psychological burden.

In our study, Female students were significantly more affected across multiple stressor domains, including academic-related stressors (AOR: 2.15, *P* < 0.001), teaching and learning-related stressors (AOR: 1.44, *P* < 0.01), social-related stressors (AOR: 1.69, *P* < 0.001), drive and desire-related stressors (AOR: 1.48, *P* = 0.006), and group activities-related stressors (AOR: 1.72, *P* < 0.001), indicating a consistent gender-based vulnerability to stress. In addition, we found that female medical students had significantly lower coping levels compared to their male counterparts (*p* = 0.002). These findings align with previous literature and may be attributed to a complex interplay of psychological, social, and cultural factors. Psychologically, females are often more self-critical and tend to internalize stress, increasing their sensitivity to academic and social pressures [32–34]. Socially and culturally, they may face greater expectations to excel academically while managing additional responsibilities or navigating gender-based challenges in clinical and academic environments [35–37]. These factors, combined with potentially less effective coping strategies and limited institutional support, may contribute to their heightened vulnerability. While some earlier studies found no gender differences in academic stress, possibly due to different curricular structures, the current integrated systems and increased academic demands may disproportionately impact female students today [22, 23].

We observed a significant increase in Academic Stress Rating (ARS) among 5th-grade students (AOR: 3.26, *p* < 0.001) compared to those in the first grade. This finding resonates with research conducted in Sudan, which indicated heightened stress levels in later academic years [20]. First-year students focus on foundational sciences in a structured setting, while final-year students face a demanding curriculum with advanced clinical work, high-stakes exams, and time-intensive rotations [20]. Moreover, continuous study over preceding years, without adequate recovery periods, may contribute to mental fatigue and diminished resilience [38]. Hoeve, Third and fourth-year students encountered higher levels of IRS, GARS, and TLRS, possibly reflecting their transition into clinical settings and increased demands for group work. Research indicates that during their medical education experience, heightened adaptation stress occurs as they shift from theoretical to practical training [39].

We observed a significant reduction in academic-related stress (ARS) among students with a monthly income between 5,000 and 10,000 L.E. (AOR: 0.64, *p* < 0.031) compared to those earning less than 5,000 L.E., likely due to reduced financial strain and better access to academic resources [40]. However, this group also demonstrated significantly lower coping abilities (*p* = 0.04), suggesting that while moderate income may alleviate academic stress, it does not necessarily enhance stress management, possibly due to unmet psychological needs or increased social and familial expectations [40–42].

Rural residence was found to significantly increase interpersonal and intrapersonal-related stressors (IRS) among medical students (AOR: 1.67, 95% CI: 1.26–2.2, *P* < 0.001), consistent with findings from a study conducted at Helwan University, Egypt. This may be attributed to cultural and social adjustment difficulties that students residing in rural areas may face when transitioning to urban areas [43]. According to Social Support Theory, strong networks of emotional, informational, and instrumental support buffer individuals from the negative effects of stress. The higher stress levels observed among female and rural students, as well as those reporting interpersonal strain, likely reflect reduced access to such support networks. This may also explain the predominant reliance on emotion-focused coping in our sample, as insufficient social support can limit students’ capacity to engage in more adaptive, problem-focused responses [44].

Students who completed the questionnaire during exam weeks exhibited higher stress levels, particularly in SRS (OR: 2.00), DRS (OR: 1.80), and GARS (OR: 1.82). Anticipation of upcoming exams (1 week to 1 month later) also increased IRS, TLRS, SRS, DRS, and GARS, reinforcing the link between exam pressure and anxiety [45]. The negative correlation between exam schedules and coping scores highlights the need for structured academic support during assessment periods.

Students with chronic illnesses had a higher likelihood of experiencing social stress SRS (OR: 1.81), likely due to the dual burden of managing health conditions and academic expectations. Similar findings in previous studies imply that chronic sickness exacerbates medical students’ stress through greater absenteeism and higher healthcare costs [3]. These findings highlight the need for targeted support and tailored interventions to address the unique challenges faced by these specific student groups.

### Strengths and limitations

This multicenter study included a large sample (984 participants) from a diverse population, and various demographics of medical students from over various universities in Egypt. It also provides comprehensive stress domains as the assessment covered multiple dimensions of stress, including academic, interpersonal, and social factors, exploring various dimensions that contribute to overall stress levels. The study highlights socioeconomic and gender disparities in stress perception, adding valuable insight into region-specific stressors. We believe that this study can offer valuable insights that can assist researchers, policymakers, and healthcare providers in formulating effective strategies to provide a healthy study environment for medical students.

On the other hand, this cross-sectional design has inherent limitations. As the study captures stress levels at a single point in time, it does not allow for the assessment of longitudinal follow-up, or causal relationships. Participants’ responses may also be subject to self-report bias, as students might underreport or overreport their stress levels. Participants were selected based on accessibility, which may introduce selection bias. The reliance on convenience sampling and online distribution via social media platforms could also lead to sampling bias, as students with limited internet access or lower online engagement may have been underrepresented. As the denominator of eligible individuals could not be precisely determined, the response rate is only an estimate based on collaborators’ outreach targets. This introduces uncertainty in participation rates and limits the ability to assess selection bias quantitatively. These factors limit the generalizability of the findings. some sensitive items (e.g., experiences of abuse) may have been underreported due to the self-administered nature of the questionnaire. Another limitation is the unbalanced distribution of participants across universities. This uneven representation may have introduced institutional bias and limited the generalizability of the findings to all Egyptian medical schools, particularly those with smaller sample sizes or differing academic environments.

Finally, the study did not include physiological indicators, such as biomarkers (e.g., cortisol levels), which could have provided objective measures of stress.

## Conclusion

This study highlights the substantial psychological burden experienced by Egyptian medical students under the newly implemented integrated (5 + 2) curriculum. Academic-related stressors emerged as the most prominent, with high to severe stress levels reported by the majority of participants, followed by considerable levels of interpersonal, social, teaching and learning, and group activity-related stressors. Factors such as female gender, advanced academic year, rural residence, lower family income, exam proximity, and chronic illness were significantly associated with heightened stress across multiple domains, while coping abilities were generally lower among female students and those from certain socioeconomic backgrounds. These findings underscore the multifactorial nature of stress in medical education and the importance of addressing both academic and non-academic influences. Targeted interventions—such as workload adjustments, clear academic expectations, anti-abuse policies, improved support systems, resilience training, and tailored assistance for vulnerable groups—are essential to promote students’ psychological well-being, enhance coping strategies, and foster a healthier learning environment.

## Supplementary Information

Supplementary Material 1.

## Data Availability

The datasets used and/or analysed during the current study are available from the corresponding author on reasonable request.

## Abbreviations

MSSQ: Medical students’ stressors questionnaire
ARS: Academic Related Stressors
IRS: Interpersonal and Intrapersonal Related Stressors
TLRS: Teaching and Learning Related Stressors
SRS: Social Related Stressors
DRS: Drive and Desire Related Stressors
GARS: Group Activities Related Stressors

## References

[1] McEWEN BS. Stress, Adaptation, and disease: allostasis and allostatic load. Ann N Y Acad Sci. 1998;840(1):33–44. 9629234 10.1111/j.1749-6632.1998.tb09546.x

[2] Folkman S, Moskowitz JT, Coping. Pitfalls and promise. Annu Rev Psychol. 2004;55(1):745–74. 14744233 10.1146/annurev.psych.55.090902.141456

[3] Dyrbye LN, Thomas MR, Shanafelt TD. Systematic review of Depression, Anxiety, And other indicators of psychological distress among U.S. And Canadian medical students. Acad Med. 2006;81(4):354–73. 16565188 10.1097/00001888-200604000-00009

[4] Nguyen T, Pu C, Waits A, Tran TD, Balhara YPS, Huynh QTV et al. Sources of stress, coping strategies and associated factors among Vietnamese first-year medical students. Forero DA, editor. PLOS ONE. 2024;19(7):e0308239. 39089322 10.1371/journal.pone.0308239 PMC11290621

[5] IsHak W, Nikravesh R, Lederer S, Perry R, Ogunyemi D, Bernstein C. Burnout in medical students: a systematic review. Clin Teach. 2013;10(4):242–5. 23834570 10.1111/tct.12014

[6] El-Gilany AH, Amr M, Hammad S. Perceived stress among male medical students in Egypt and Saudi Arabia: effect of sociodemographic factors. Ann Saudi Med. 2008;28(6):442–8. 10.5144/0256-4947.2008.442. PMID: 19011321; PMCID: PMC6074256. 10.5144/0256-4947.2008.442 PMC6074256 19011321

[7] Heinen I, Bullinger M, Kocalevent RD. Perceived stress in first year medical students - associations with personal resources and emotional distress. BMC Med Educ. 2017;17(1):4. 28056972 10.1186/s12909-016-0841-8 PMC5216588

[8] Badrawi N, Hosny S, Ragab L, Ghaly M, Eldeek B, Tawdi AF, et al. Radical reform of the undergraduate medical education program in a developing country: the Egyptian experience. BMC Med Educ. 2023;23(1):143. 36869307 10.1186/s12909-023-04098-3 PMC9983512

[9] الجهاز. المركزي للتعبئة العامة والإحصاء. [cited 2025 Dec 5]. Available from: https://www.capmas.gov.eg/home

[10] وزارة التعليم العالي والبحث العلمي. 2018 [cited 2025 Dec 5]. الرئيسية. Available from: https://mohesr.gov.eg/

[11] Yusoff MSB, Rahim AFA, Yaacob MJ. Medical Student Stressor Questionnaire. 2013 [cited 2025 Nov 30]. Available from: 10.1037/t15334-000

[12] Yusoff MSB. A Confirmatory Factor Analysis Study on the Medical Student Stressor Questionnaire among Malaysian medical students. Educ Med J. 2011 June 18 [cited 2025 Nov 30];3(1). Available from: http://www.eduimed.com/index.php/eimj/article/view/95

[13] Hamby, Sherry, Grych, John, Banyard, Victoria. Coping Scale. 2015. 10.13140/RG.2.1.3094.0001.

[14] Manitz J, Hempelmann M, Kauermann G, Kuechenhoff H, Shao S, Oberhauser C et al. samplingbook: Survey Sampling Procedures. 2021 [cited 2025 Nov 30]. Available from: https://cran.r-project.org/web/packages/samplingbook/index.html

[15] R: The R Project for Statistical Computing. [cited 2025 Apr 15]. Available from: https://www.r-project.org/

[16] Muhamad Saiful Bahri Yusoff. Ahmad Fuad Abdul Rahim. The medical student stressor questionnaire (MSSQ) manual. Kubang Kerian. Kelantan: Universiti Sains Malaysia; 2010.

[17] Elshafie S. Reform strategies of medical education in Egypt. Fayoum Univ Med J. 2018;1(1):24–7.

[18] Ebrahim OS, Sayed HA, Rabei S, Hegazy N. Perceived stress and anxiety among medical students at Helwan university: A cross-sectional study. J Public Health Res. 2024;13(1):22799036241227891. 38313630 10.1177/22799036241227891 PMC10838489

[19] Caplan RD. Person-environment fit theory and organizations: commensurate dimensions, time perspectives, and mechanisms. J Vocat Behav. 1987;31(3):248–67.

[20] Ragab EA, Dafallah MA, Salih MH, Osman WN, Osman M, Miskeen E, et al. Stress and its correlates among medical students in six medical colleges: an attempt to understand the current situation. Middle East Curr Psychiatry. 2021;28(1):75.

[21] Al-Shahrani MM, Alasmri BS, Al-Shahrani RM, Al-Moalwi NM, Al Qahtani AA, Siddiqui AF. The Prevalence and Associated Factors of Academic Stress among Medical Students of King Khalid University: An Analytical Cross-Sectional Study. Healthcare. 2023;11(14):2029. 37510470 10.3390/healthcare11142029 PMC10378871

[22] Okoye OC. Perceived stress and stressors among undergraduate medical students of a Nigerian institution. Malawi Med J. 2022;34(4):245–51. 38125774 10.4314/mmj.v34i4.4 PMC10645827

[23] Stress and Coping Strategies among Medical Students in Zagazig University - A Prospective Cohort Study. Egypt J Community Med. 2021;39(2):1–12.

[24] Sweller J. Cognitive load during problem solving: effects on learning. Cogn Sci. 1988;12(2):257–85.

[25] Liu Z, Xie Y, Sun Z, Liu D, Yin H, Shi L. Factors associated with academic burnout and its prevalence among university students: a cross-sectional study. BMC Med Educ. 2023;23(1):317. 37149602 10.1186/s12909-023-04316-y PMC10163855

[26] Premnath A, Sivan S, Velayudhan R, Ms S, Tm R. Effect of a Stress Reduction Programme on Academic Stress and Coping Skills of First Year Medical Students. Kerala J Psychiatry. 2020 Apr 11 [cited 2025 Dec 4];33(1). Available from: https://kjponline.com/index.php/kjp/article/view/187/255

[27] El Nouiri A, El Kassem S, Al Maaz Z, Alhajj Y, Al Moussawi A, El Yaman A, et al. Prevalence and characteristics of medical student mistreatment in Lebanon. Int J Public Health. 2024;69:1606710. 39027015 10.3389/ijph.2024.1606710 PMC11254615

[28] Elghazally NM, Atallah AO. Bullying among undergraduate medical students at Tanta University, egypt: a cross-sectional study. Libyan J Med. 2020;15(1):1816045. 32877320 10.1080/19932820.2020.1816045 PMC7646535

[29] Kaur G, Peng K, Urwin R, Westbrook JI, McMullan RD. Is there a relationship between medical student mistreatment and specialty choice and career intentions? A systematic review. Med Sci Educ. 2025;35(3):1777–86. 40625953 10.1007/s40670-025-02340-9 PMC12228901

[30] Zhang H, li, Liu F, Lang Hjuan. The relationship between role ambiguity and anxiety in intensive care unit nurses: the mediating role of emotional intelligence. Intensive Crit Care Nurs. 2024;81:103597. 38029677 10.1016/j.iccn.2023.103597

[31] Deci EL, Ryan RM. Intrinsic Motivation and Self-Determination in Human Behavior [Internet]. Boston, MA: Springer US; 1985 [cited 2025 Dec 5]. Available from: http://link.springer.com/10.1007/978-1-4899-2271-7

[32] Franzoi SL, Vasquez K, Sparapani E, Frost K, Martin J, Aebly M. Exploring body comparison tendencies: women are Self-Critical whereas men are Self-Hopeful. Psychol Women Q. 2012;36(1):99–109.

[33] Kopala-Sibley DC, Klein DN, Perlman G, Kotov R. Self-criticism and dependency in female adolescents: prediction of first onsets and disentangling the relationships between personality, stressful life events, and internalizing psychopathology. J Abnorm Psychol. 2017;126(8):1029–43. 29154564 10.1037/abn0000297 PMC5728430

[34] Farhane-Medina NZ, Luque B, Tabernero C, Castillo-Mayén R. Factors associated with gender and sex differences in anxiety prevalence and comorbidity: A systematic review. Sci Prog. 2022;105(4):00368504221135469. 36373774 10.1177/00368504221135469 PMC10450496

[35] Chaplin TM, Hong K, Bergquist K, Sinha R. Gender differences in response to emotional stress: an assessment across Subjective, Behavioral, and physiological domains and relations to alcohol craving. Alcohol Clin Exp Res. 2008;32(7):1242–50. 18482163 10.1111/j.1530-0277.2008.00679.x PMC2575018

[36] Alfonso S, Diniz AM, Deaño M, Tellado F, García-Señorán M, Conde Á, et al. Gender, planning, and academic expectations in first-year higher education students: testing two alternative mediation models. Psicol Reflex E Crítica. 2020;33(1):5. 10.1186/s41155-020-00142-z PMC7206470 32383059

[37] Begum V, Anwer Arshi T, Said Arman A, Saleem Butt A, Latheef S. A study on work-family life imbalance among women administrators in UAE higher education institutions. Heliyon. 2024;10(6):e28286. 38533041 10.1016/j.heliyon.2024.e28286 PMC10963610

[38] Stress and Burnout among Egyptian Undergraduate Medical Students. Egypt J Community Med. 2021;39(3):93–103.

[39] Yusoff MSB, Abdul Rahim AF, Yaacob MJ. Prevalence and sources of stress among universiti sains Malaysia medical students. Malays J Med Sci MJMS. 2010;17(1):30–7. 22135523 PMC3216143

[40] Bergmann C, Muth T, Loerbroks A. Medical students’ perceptions of stress due to academic studies and its interrelationships with other domains of life: a qualitative study. Med Educ Online. 2019;24(1):1603526. 31007152 10.1080/10872981.2019.1603526 PMC6493308

[41] Kothari V, George N, Hamid O. Provision of mental health support for medical students. Adv Med Educ Pract. 2018;9:925–6. 30588147 10.2147/AMEP.S184571 PMC6298872

[42] Deng Y, Cherian J, Khan NUN, Kumari K, Sial MS, Comite U, et al. Family and academic stress and their impact on students’ depression level and academic performance. Front Psychiatry. 2022;13:869337. 35782431 10.3389/fpsyt.2022.869337 PMC9243415

[43] Landsman MJ, Rathman D. Rural challenges in social work regulation. Res Soc Work Pract. 2023;33(1):121–31.

[44] Cohen S, Wills TA. Stress, social support, and the buffering hypothesis. Psychol Bull. 1985;98(2):310–57. 3901065

[45] Abdulghani HM, AlKanhal AA, Mahmoud ES, Ponnamperuma GG, Alfaris EA. Stress and its effects on medical students: A Cross-sectional study at a college of medicine in Saudi Arabia. J Health Popul Nutr. 2011;29(5):516–22. 22106758 10.3329/jhpn.v29i5.8906 PMC3225114

[46] Guideline for good clinical practice (GCP) E6(R3)EMA/CHMP/ICH/135/1995EMA/CHMP/ICH/135/1995.

[47] Goodyear MDE, Krleza-Jeric K, Lemmens T. The declaration of Helsinki. BMJ 2007 Sept;335(7621):624–5. 10.1136/bmj.39339.610000.BE PMC1995496 17901471

