# Supplementary material for: Stressors and coping strategies among Egyptian medical students under the integrated curriculum: a multicenter cross-sectional study
Source: BMC Med Educ. 2026 Jan 16;26:118. doi: 10.1186/s12909-025-08487-8 (PMC12825207; doi:10.1186/s12909-025-08487-8)
Supplement: Supplementary file 1 — Supplementary Material 1. [file 12909_2025_8487_MOESM1_ESM.docx]

***Supplementary Table 1:*** *Distribution of participating medical students by university in Egypt (N = 984)*

| **Variable** | **n (%)** |
| --- | --- |
| **University** |  |
| Al-Azhar University Faculty of Medicine | 160 (16.3%) |
| Cairo University Kasr Alainy School of Medicine | 116 (11.8%) |
| Assiut University Faculty of Medicine | 115 (11.7%) |
| Beni Suef University Faculty of Medicine | 108 (11.0%) |
| Mansoura University Faculty of Medicine | 88 (8.9%) |
| Alexandria University Faculty of Medicine | 65 (6.6%) |
| South Valley University Qena Faculty of Medicine | 53 (5.4%) |
| Kafrelsheikh university faculty of medicine | 49 (5.0%) |
| Aswan University Faculty of Medicine | 42 (4.3%) |
| Ain Shams University El-Demerdash Faculty of Medicine | 37 (3.8%) |
| Tanta University Faculty of Medicine | 34 (3.5%) |
| Benha University Faculty of Medicine | 23 (2.3%) |
| Port Said University Faculty of Medicine | 19 (1.9%) |
| Menoufia University Faculty of Medicine | 16 (1.6%) |
| Zagazig University Faculty of Medicine | 16 (1.6%) |
| Badr University in Cairo School of Medicine | 6 (0.6%) |
| Minia University Faculty of Medicine | 6 (0.6%) |
| Helwan University Faculty of Medicine | 4 (0.4%) |
| Nahda University in Beni Suef Faculty of Medicine | 4 (0.4%) |
| Fayoum University Faculty of Medicine | 3 (0.3%) |
| Modern University for Technology and Information Faculty of Medicine | 3 (0.3%) |
| Sohag University Faculty of Medicine | 3 (0.3%) |
| Arab Academy for Science, Technology and Maritime Transport College of Medicine | 2 (0.2%) |
| Damietta University Faculty of Medicine | 2 (0.2%) |
| Merit University Faculty of Medicine | 2 (0.2%) |
| Misr University for Science and Technology College of Medicine | 2 (0.2%) |
| New Mansoura University Faculty of Medicine | 2 (0.2%) |
| Delta University for Science and Technology Faculty of Medicine | 1 (0.1%) |
| Horus University Faculty of Medicine | 1 (0.1%) |
| October 6 University Faculty of Medicine | 1 (0.1%) |
| Suez Canal University Faculty of Medicine | 1 (0.1%) |

***Table 2:*** *GVIFs of the final ARS multivariable model*

| Variable | GVIF | Df | GVIF^(1/(2*Df)) |
| --- | --- | --- | --- |
| Sex | 1.076 | 1 | 1.037 |
| Residence | 1.033 | 1 | 1.016 |
| Grade | 1.231 | 4 | 1.026 |
| Monthly income | 1.084 | 2 | 1.020 |
| Chronic disease | 1.011 | 1 | 1.006 |
| Next exam | 1.147 | 2 | 1.035 |

***Table 3:*** *GVIFs of the final IRS multivariable model*

| Variable | GVIF | Df | GVIF^(1/(2*Df)) |
| --- | --- | --- | --- |
| Sex | 1.097 | 1 | 1.048 |
| Residence | 1.032 | 1 | 1.016 |
| Grade | 1.240 | 4 | 1.027 |
| Monthly income | 1.118 | 2 | 1.028 |
| Chronic disease | 1.017 | 1 | 1.009 |
| Next exam | 1.151 | 2 | 1.036 |

***Table 4:*** *GVIFs of the final TLRS multivariable model*

| Variable | GVIF | Df | GVIF^(1/(2*Df)) |
| --- | --- | --- | --- |
| Sex | 1.100 | 1 | 1.049 |
| Residence | 1.034 | 1 | 1.017 |
| Grade | 1.248 | 4 | 1.028 |
| Monthly income | 1.109 | 2 | 1.026 |
| Chronic disease | 1.016 | 1 | 1.008 |
| Next exam | 1.139 | 2 | 1.033 |

***Table 5:*** *GVIFs of the final SRS multivariable model*

| Variable | GVIF | Df | GVIF^(1/(2*Df)) |
| --- | --- | --- | --- |
| Sex | 1.108 | 1 | 1.053 |
| Residence | 1.037 | 1 | 1.018 |
| Grade | 1.252 | 4 | 1.028 |
| Monthly income | 1.116 | 2 | 1.028 |
| Chronic disease | 1.018 | 1 | 1.009 |
| Next exam | 1.135 | 2 | 1.032 |

***Table 6:*** *GVIFs of the final DRS multivariable model*

| Variable | GVIF | Df | GVIF^(1/(2*Df)) |
| --- | --- | --- | --- |
| Sex | 1.106 | 1 | 1.051 |
| Residence | 1.040 | 1 | 1.020 |
| Grade | 1.255 | 4 | 1.029 |
| Monthly income | 1.109 | 2 | 1.026 |
| Chronic disease | 1.015 | 1 | 1.007 |
| Next exam | 1.134 | 2 | 1.032 |

***Table 7:*** *GVIFs of the final GARS multivariable model*

| Variable | GVIF | Df | GVIF^(1/(2*Df)) |
| --- | --- | --- | --- |
| Sex | 1.100 | 1 | 1.049 |
| Residence | 1.043 | 1 | 1.021 |
| Grade | 1.270 | 4 | 1.030 |
| Monthly income | 1.110 | 2 | 1.027 |
| Chronic disease | 1.016 | 1 | 1.008 |
| Next exam | 1.151 | 2 | 1.036 |

***Table 8:*** *GVIFs of the final coping multivariable model*

| Variable | GVIF | Df | GVIF^(1/(2*Df)) |
| --- | --- | --- | --- |
| Sex | 1.093 | 1 | 1.045 |
| Residence | 1.048 | 1 | 1.023 |
| Grade | 1.246 | 4 | 1.028 |
| Monthly income | 1.111 | 2 | 1.027 |
| Chronic disease | 1.015 | 1 | 1.007 |
| Next exam | 1.144 | 2 | 1.034 |
